# Chin Ptosis: Etiology, Classification, Diagnosis and Surgical Management—A Literature Review

**DOI:** 10.3390/jcm15208175

**Published:** 2026-10-21

**Authors:** Agnieszka Rolek, Rafał Pokrowiecki

**Affiliations:** 1 Independent Researcher, ul. R. Dmowskiego 1A/43, 50-203 Wroclaw, Poland; 2 Independent Researcher, Woronicza 31/33, 02-640 Warsaw, Poland

**Keywords:** chin ptosis, witch’s chin, mentalis muscle, genioplasty, chin rejuvenation, facial aging

## Abstract

**Background/Objectives:** Chin ptosis is a heterogeneous deformity characterized by inferior displacement of the presymphyseal soft tissues and may reflect abnormalities of skeletal support, the mentalis muscle, the submental crease, the soft-tissue envelope, or facial animation. This narrative review aimed to synthesize the anatomy, etiology, classification, diagnostic assessment, and the management of chin ptosis and to translate the available evidence into a component-based clinical framework. **Methods:** MEDLINE via PubMed was searched from inception to 6 October 2026 and yielded 62 records. Targeted Google Scholar searches and backward citation searching identified 22 additional unique sources not retrieved by the PubMed strategy. Forty-two publications were included. Anatomical studies, technical reports, clinical series, comparative studies, reviews, and relevant book chapters were evaluated. Evidence was classified as direct when it concerned diagnosed chin ptosis and as indirect when it concerned related conditions such as chin retrusion, macrogenia, facial aging, or mentalis hyperactivity. **Results:** The four-category classification of illusory, developmental, iatrogenic, and dynamic chin ptosis derives from the framework of Garfein and Zide and was retained with additional clarification. The categories are not mutually exclusive. Diagnosis should integrate history, examination at rest and during animation, skeletal and dental assessment, standardized photography or video, reproducible soft-tissue measurements, and patient-reported outcomes. Structural mentalis detachment generally requires anatomical reattachment, whereas crease abnormalities, skeletal mismatch, redundant soft tissue, or generalized cervicofacial laxity require component-specific correction. Nonsurgical treatments mainly address projection deficiency or muscular hyperactivity and should not be presented as definitive treatment for true structural ptosis. **Conclusions:** Mechanism-based diagnosis and treatment selection are essential. Current evidence remains dominated by technical reports and small retrospective series; standardized diagnostic criteria, complication reporting, and comparative prospective studies are needed.

## 1. Introduction

### 1.1. Background

The chin plays a pivotal role in defining lower facial esthetics, contributing significantly to facial harmony, profile balance, and the conveyance of emotional expression. Its anatomical complexity, involving bony structures such as the mandible and soft tissues, including the mentalis muscle and overlying skin, means that alterations in this region can profoundly impact facial appearance. Chin ptosis is explained as the descent of the soft tissues of the chin below the lower contour of the mandible. Many factors such as aging, developmental issues, or surgical complications can lead to this condition, it is highly relevant in the context of facial rejuvenation, orthognathic surgery, and chin implant revisions, where failure of recognition may lead to suboptimal outcomes or postoperative deformities [1,2]. Despite its clinical importance, ptosis of the chin pad remains a complex and multifactorial problem that necessitates a clear understanding of its anatomy, pathophysiology, and classification to guide effective management [2,3]. Various surgical techniques, including soft tissue suspension with sutures, muscle resuspension, and bone contouring, have been developed, aiming to restore a youthful and harmonious facial profile. Recent innovations, such as the use of surgical nets or minimally invasive suture suspension, offer effective and less invasive options with high patient satisfaction and low complication rates [2,4,5].

### 1.2. Aim

This article aims to provide a comprehensive overview of current knowledge on chin ptosis, with particular emphasis on its anatomy, pathophysiology, etiology, classification, clinical assessment, and available treatment options. A thorough understanding of its multifactorial etiology and anatomical considerations is imperative for achieving optimal esthetic and functional outcomes in patients presenting with reported condition.

## 2. Materials and Methods

A structured narrative review was conducted to summarize the anatomy, etiology, classification, diagnosis, and management of chin ptosis. The review was prepared with reference to the Scale for the Assessment of Narrative Review Articles (SANRAs) [6]. MEDLINE via PubMed was searched from database inception to 6 October 2026. Targeted Google Scholar searches and backward citation searching of eligible publications and relevant reviews were used to locate additional sources, particularly historically important descriptions of operative techniques and sources not indexed by the PubMed strategy. No restrictions were placed on year of publication because many established concepts and procedures were introduced in older literature.

The core concepts were “chin ptosis”, “ptotic chin”, “witch’s chin”, “senile chin deformity”, “mentalis muscle”, “chin pad”, and “submental crease”. These were combined with terms relating to anatomy, etiology, pathogenesis, classification, diagnosis, treatment, mentalis reattachment or resuspension, genioplasty, chin implants, facelift, and neck lift. The exact PubMed strategy and the supplementary source-identification methods are reported in the Table 1. Google Scholar was used as a targeted discovery tool rather than as a source of a reproducible total hit count; only unique sources that contributed to the final synthesis were logged.

Eligible publications addressed at least one of the following: anatomy or function of the mentalis muscle; etiology or classification of chin ptosis; clinical or imaging assessment; differential diagnosis; prevention; surgical or nonsurgical treatment; outcomes; or complications. Anatomical studies, technical descriptions, case reports, case series, retrospective or prospective clinical studies, comparative studies, narrative or systematic reviews, and relevant book chapters were considered. Publications were excluded when they had no clinically relevant relationship to the chin soft-tissue envelope, duplicated another report, lacked an accessible English translation, or addressed general facial aging or genioplasty without extractable information relevant to chin position, mentalis integrity, or soft-tissue support.

All 62 PubMed titles and abstracts were screened and their relevance to the prespecified eligibility criteria were assessed. Twenty PubMed-indexed sources were retained. Targeted Google Scholar searching and backward citation searching contributed 22 additional unique sources not retrieved by the PubMed strategy, resulting in 42 publications in the narrative synthesis. The remaining 42 PubMed records were not retained because they had an unrelated clinical or anatomical focus, lacked extractable or nonredundant information relevant to chin ptosis, or were retracted, comment-only, or duplicate items. Only unique supplementary sources included in the synthesis were logged; therefore, no total Google Scholar denominator or separate duplicate-removal count was reported. The selection process is summarized in Figure 1.

Data were extracted on study design, sample size, anatomical target, patient selection, type of ptosis or related deformity, intervention, follow-up, outcomes, complications, and limitations. Evidence was categorized as direct when the study population was diagnosed with chin ptosis or witch’s chin and as indirect when the evidence concerned a related condition—such as chin retrusion, macrogenia, general cervicofacial aging, or isolated mentalis hyperactivity—from which only a specific anatomical or therapeutic principle could be inferred. Because the evidence was heterogeneous and consisted predominantly of anatomical studies, technical reports, and small retrospective series, no meta-analysis or formal risk-of-bias assessment was performed. Findings were synthesized narratively and compared according to anatomical target, indication, outcome, complication profile, and directness of evidence.

## 3. Relevant Chin Anatomy

The chin is a composite anatomical unit in which mandibular projection, periosteum, the paired mentalis muscles, subcutaneous fat, retaining attachments, and skin function as an integrated support system. The mentalis originates from the anterior mandible inferior to the attached gingiva of the mandibular incisors and inserts into the deep dermis of the chin pad. Its contraction elevates and protrudes the lower lip, wrinkles the chin skin, and contributes to lip competence. Consequently, an operation that changes only bone or skin can alter the lower lip–chin relationship if the mentalis attachment and the volume of the overlying envelope are not preserved [2,7,8].

The mentalis does not have a uniform morphology. Cadaveric and ultrasonographic studies have described a dome-shaped type A muscle and a flatter, sparsely developed type B muscle. Type A can be subdivided into A-1, in which the bilateral bellies merge in the midline, and A-2, in which they remain separated. Choi et al. found the muscle predominantly 5–10 mm lateral to the midsagittal line and 20–30 mm inferior to the intercheilion line; at points 5 mm lateral to the soft-tissue pogonion, the mean depth from the skin was 6.7 mm and the mean muscle thickness was 4.0 mm [9]. These measurements are particularly relevant to ultrasound-guided assessment and botulinum toxin injection but also illustrate why a uniform depth of surgical fixation cannot be assumed.

Anatomical variation has practical implications for surgical planning. A fused A-1 pattern may permit continuous midline support, whereas an A-2 pattern requires symmetric identification and fixation of two separate bellies to avoid differential tension. In a thin type B muscle, the limited tissue available for sutures may reduce fixation security and increase susceptibility to contour irregularity or recurrent descent. Conversely, excessive central reefing of a thick or overlapping muscle can deepen the labiomental fold, cause bunching or dimpling, and distort lower-lip position. These associations are anatomically plausible but have not yet been validated in comparative outcome studies; they should therefore guide individualized dissection and fixation rather than be interpreted as proven predictors of recurrence [7,8,9] (Figure 2).

The vascularity and integrity of the proximal muscle must be respected during intraoral surgery. Wide degloving, repeated dissection, transection close to the origin, or imprecise closure may leave the mentalis shortened, scarred, devascularized, or fixed in an inferior position. Revision surgery is therefore less predictable than primary anatomical repair. The lateral extent of dissection should also be controlled to reduce injury to the mental nerves, while the amount and vector of cephalic suspension should restore the preoperative lower lip–chin relationship without producing overcorrection. When muscle continuity is uncertain, particularly after multiple operations, palpation, dynamic examination, and selective ultrasonography may assist preoperative planning [2,7,9,10].

## 4. Origin and Classification of Chin Ptosis

### 4.1. Pathogenesis of Chin Ptosis

Historically, early studies on chin ptosis primarily focused on age-related changes and edentulism, particularly the loss of mandibular incisors, as the main contributing factors to the development of this deformity. At that time, the pathogenesis of chin ptosis was widely believed to be related primarily to age-related changes [11].

In 1987, H. George Brennan et al. described the pathogenesis of chin ptosis primarily as a consequence of age-related changes and tooth loss, particularly the loss of frontal mandibular teeth. According to their concept, progressive alveolar bone resorption results in inferior displacement of the mentalis muscle attachment, leading to a loss of support for the pre-symphyseal fibrofatty tissues and subsequent soft tissue descent. In addition, resorption of the mandibular symphysis and downward displacement of the chin pad contribute to reduced chin projection and an inferior rather than anterior orientation of the soft tissues. The authors also attributed the deepening of the submental crease to soft tissue ptosis and accumulation of adipose tissue within this anatomically predisposed region, while redundant submental skin was considered a natural consequence of the aging process [12].

Today, this mechanism is recognized as only one of several etiological pathways leading to chin ptosis. Currently, four primary categories of chin ptosis are identified: illusory, developmental, iatrogenic, and dynamic ptosis. Understanding these classifications allows surgeons to optimize treatment strategies, improve functional outcomes, and achieve superior esthetic results [2,13,14].

### 4.2. Terminology and Definitions: Chin Ptosis Versus Witch’s Chin

It is important to clarify the distinction between commonly used term “witch’s chin” and properly identified chin ptosis. The term witch’s chin has traditionally been used to describe a characteristic chin deformity involving ptosis of the premental soft tissues, a pronounced submental crease, and frequently reduced underlying bony projection, producing a distinctive downward and forward displacement of the chin. González–Ulloa first described this condition in 1972 as a form of chin ptosis; however, subsequent authors have emphasized that this terminology does not adequately distinguish between different types of chin deformity [3,15].

Clinically, witch’s chin is characterized by a prominent chin soft-tissue pad that extends both anteriorly and inferiorly, creating the projecting and drooping appearance commonly associated with age-related or senile changes. The soft-tissue pad descends below the inferior mandibular border while retaining its characteristic forward projection [3,13].

In contrast, true chin ptosis refers to inferior displacement of the presymphyseal soft tissues below the lower border of the mandible without the anterior projection characteristic of witch’s chin. Thus, the deformity is primarily manifested as downward descent of the chin pad rather than its protrusion [3] (Figure 3).

### 4.3. Classification

The four-category system used in this review is not a new classification proposed by the present authors. It was introduced by Garfein and Zide, who distinguished illusory, developmental, iatrogenic, and dynamic chin ptosis [2]. The present review retains this established etiological–clinical framework and supplements it with a component-based analysis of skeletal support, mentalis attachment and function, submental crease morphology, soft-tissue volume, skin and platysmal laxity, and facial animation. The categories describe dominant mechanisms rather than severity grades and are not necessarily mutually exclusive; mixed deformities should be recorded by identifying each contributing component.

#### 4.3.1. Developmental (Non-Iatrogenic Support-Loss/Age-Associated) Chin Ptosis

In the original classification, “developmental” does not denote a congenital anomaly. It refers to progressive non-iatrogenic loss of skeletal and dental support, including a result of bone resorption secondary to teeth loss, periodontal disease, ill-fitting dentures, or dental bone removal. It is not related to the surgery performed in the chin area (sliding genioplasty, implant placement) which includes muscle transection during the surgery, especially through mandibular vestibular approach [16]. In DCP, after teeth extraction, the alveolar ridge undergoes resorption because the mechanical strain stimulus on the bone is significantly reduced. The removal of the tooth and periodontal ligament decreases the bending strains in the mandible, leading to a lower strain stimulus that is insufficient to maintain bone mass. Consequently, bone resorption occurs mainly at the external bone surface to restore the strain levels to pre-extraction conditions [17]. According to studies by Antje Tallgren the wearing of complete dentures leads to a continuing reduction in the residual alveolar ridges, particularly marked on the lower ridge. Longitudinal research covering up to 25 years revealed that the mean reduction in anterior height of the mandibular ridge is approximately four times greater than that of the maxillary ridge [11]. The loss of mandibular vertical height leads to inferior displacement of the mentalis muscle origin and chin ptosis. This condition is irreversible but may be stabilized by implants and proper denture adjustment to prevent further bone loss [2]. The historical term is retained to remain consistent with the source classification; however, it should not be used interchangeably with generalized senile laxity. Aging may contribute independently through skin redundancy, fat redistribution, and platysmal laxity even when substantial alveolar resorption is absent. Thus, age-related change is better regarded as a modifier that may coexist with developmental support loss, illusory crease deformity, or another ptosis subtype.

#### 4.3.2. Illusory Chin Ptosis

Illusory Chin Ptosis is characterized by the perceived descent of the chin soft tissues, often due to an exaggerated submental crease extending cephalad over the inferior mandibular border. Despite the appearance, the lip position, intraoral sulcus, and movement are normal. It commonly occurs in patients without prior surgery [2]. Several methods for correcting a deep submental crease associated with Witch’s Chin deformity have been described in the literature. In 1982, Burke described a technique involving deepithelialization of a transverse ellipse of submental skin that includes the submental fold. The skin anterior to this ellipse is undermined, and the deepithelialized skin is rolled onto itself to create soft tissue fill in the area of the deficit. The ellipse is closed then [3,18]. In the same year Peterson developed similar technique incorporating a full-thickness incision at the posterior edge, undermining beneath the ellipse, and fixation of the dermal flap to the platysma, along with chin implant placement [3,19]. In 1996 Lesavoy et al. found that Peterson’s method often led to prominent dog ears upon closure of the skin ellipse. To address this, they modified Peterson’s technique by marking a horizontal ellipse with the submental fold as its long axis, deepithelializing it, and then creating two divergent, anteriorly based, triangular flaps of deepithelialized skin. The medial portion of the deepithelialized skin was excised, and the flaps were sutured to each other convergently. The deepithelialized flaps were then sutured to the underlying platysma muscle as a single unit. The posterior skin was undermined to the level of the hyoid bone, advanced anteriorly, and approximated to the anterior edge of the ellipse. Their studies show that this modification helped to avoid dog ears and improved closure precision. In 1992, Feldman et al. proposed that in some cases, the submental crease appears deeper than it actually is, and the soft tissue defect is illusory. He suggested that for these patients, local flaps are unnecessary, and correction can be achieved by excising a transverse ellipse of skin and subcutaneous tissue encompassing the submental crease [3,20]. In their 1999 study, Stelnicki et al. recommended a combined surgical approach for the treatment of illusory chin ptosis, integrating previously described techniques. Their method included excision or deepithelialization of the deep submental crease followed by a pants-over-vest closure, lateral undermining to facilitate soft-tissue redraping, and, when indicated, augmentation with a de-epithelialized dermal flap or a small chin implant [21].

#### 4.3.3. Iatrogenic Chin Ptosis

Iatrogenic chin ptosis is a condition that arises primarily from surgical interventions involving the chin, particularly those performed via an intraoral approach. This approach can compromise the proximal mentalis muscle by devascularizing its origin, leading to a downward drift of the muscle and soft tissue descent below the mandibular border [2]. Failure to properly reattach the mentalis muscle after procedures such as genioplasty or chin implant placement and removal is a significant contributing factor, resulting in weakened muscle support and subsequent ptosis [2]. Additionally, scarring and bone resorption following implant removal can leave the soft tissues unsupported, exacerbating the deformity [3]. The surgical correction of iatrogenic chin ptosis typically involves resuspension of the mentalis muscle to its anatomically correct position, often utilizing intraosseous anchors for stable reattachment, combined with wide soft tissue undermining to allow adequate redraping [2,3]. This condition underscores the importance of meticulous surgical technique and the preservation of mentalis muscle integrity during chin surgeries to prevent postoperative ptosis [22]. The importance of precise mentalis muscle reattachment and resuspension in preventing and correcting iatrogenic chin ptosis has been increasingly emphasized in the literature. In 2001, Chaushu et al. [23] evaluated postoperative soft-tissue changes in patients undergoing advancement genioplasty with and without accurate reapproximation of the mentalis muscle The most pronounced differences between the groups were observed in the vertical parameters [23]. In patients who underwent precise mentalis reattachment, lower lip length was generally maintained or increased, whereas in those without precise reattachment it tended to remain unchanged or decrease, resulting in a mean vertical difference of approximately 6 mm between the groups. Accordingly, lower incisor exposure at rest remained stable or decreased following surgery in the reattachment group, while increased exposure was observed in the control group. Similar differences were found in the vertical position of the soft-tissue supramentale: it remained essentially unchanged when the mentalis muscle was accurately reattached, whereas an average inferior displacement of 2.14 mm occurred in the control group. Clinically, this downward migration of the soft tissues manifested as chin ptosis, providing evidence that inadequate restoration of the mentalis muscle attachment following genioplasty may contribute directly to postoperative descent of the chin soft-tissue [23]. Zide and Boutros subsequently emphasized the anatomical variability of the mentalis muscle and advocated reefing and fixation of the muscle in a higher position to restore and maintain the appropriate labiomental sulcus and lower lip–chin relationship [7]. Garfein and Zide later systematized these principles specifically for iatrogenic chin ptosis, recommending wide release of the malpositioned soft tissues followed by cephalic resuspension of the mentalis muscle close to its original attachment, with stable fixation to prevent recurrent inferior migration of the chin pad [2]. More recently, the emphasis has shifted from secondary correction toward preservation of the mentalis attachment during the primary procedure. Zhang et al. demonstrated that a no-degloving genioplasty technique, designed to minimize disruption of the soft-tissue attachments, resulted in less postoperative soft-tissue displacement and no cases of witch’s chin deformity in their no-degloving group, compared with six cases in the traditional degloving group [10].

The clinical relevance of mentalis dysfunction following advancement genioplasty and the need for its surgical correction were further highlighted by Clark and Baur in 2004. The case they described demonstrates that inadequate restoration of the mentalis muscle to its native attachment on the anterior mandible may result in abnormal muscle contraction, producing visible bunching, irregularity, or dimpling of the overlying chin skin. Surgical repositioning and reattachment of the mentalis to the anterior mandibular surface can improve chin ptosis; however, in some patients, a persistent prominence or localized bulging of the muscle may remain despite appropriate reapproximation. Persistent hyperactivity of the mentalis muscle has been suggested as a potential contributing factor to this residual deformity [24].

Collectively, these findings indicate an evolution from secondary mentalis resuspension toward precise primary reattachment and, ultimately, preservation of the native mentalis attachment whenever possible, thereby maintaining the structural support of the lower lip and chin and reducing the risk of iatrogenic chin ptosis.

#### 4.3.4. Dynamic Chin Ptosis

Dynamic chin ptosis is a distinct form of chin deformity in which the soft-tissue chin pad becomes flattened against the underlying mandibular symphysis and displaced inferiorly during facial animation, particularly during smiling. The condition may be developmental or acquired and is most evident in patients with a predominantly horizontal smile, in whom insufficient elevation in the oral commissures allows the horizontal vector of the risorius muscle to predominate. As a result, the chin pad is compressed against the bony prominence of the symphysis and displaced below the inferior mandibular border [2]. The deformity may also be induced or aggravated by surgical procedures, particularly reduction in the mandibular symphysis, which alters the underlying skeletal support of the chin soft tissues [2]. Because both skeletal morphology and soft-tissue dynamics contribute to its development, treatment should be tailored to the underlying mechanism. Torrealba et al. described a surgical modification for dynamic chin ptosis associated with reduction in the mandibular symphysis, emphasizing that correction of the submental crease alone addresses only the soft-tissue component and may therefore be insufficient when an altered skeletal framework contributes to the deformity [14]. The importance of muscular function in determining chin position and contour is further supported by Hsu and Frankel, who evaluated patients with a high-riding, hyperactive mentalis muscle. Following chemodenervation of the mentalis with onabotulinumtoxinA, the soft-tissue pogonion shifted significantly inferiorly in most patients, accompanied by an inferior redistribution of soft-tissue volume, a more rounded chin contour, and reduced muscular tension [25]. Although their study did not specifically investigate dynamic chin ptosis, these findings provide additional evidence that mentalis activity can substantially influence the vertical position and morphology of the chin soft-tissue envelope. Conversely, excessive or improperly distributed weakening of the mentalis and diffusion of botulinum toxin into adjacent lower-lip muscles may produce undesirable functional and esthetic changes, including lower-lip ptosis, oral incompetence, asymmetry, or paradoxical chin bulging [26,27].

Mixed phenotypes are common. For example, an edentulous patient may have developmental loss of skeletal support together with a deep crease and age-related skin redundancy, whereas a patient after reduction genioplasty may have both iatrogenic mentalis displacement and dynamic worsening during smiling. Classification should therefore identify the predominant mechanism but treatment planning should document all abnormal components rather than force every patient into a single exclusive category (Table 2).

## 5. Diagnostic Assessment

### 5.1. History and Clinical Examination

Assessment should begin with the timing and circumstances of onset. Previous genioplasty, intraoral implant placement or removal, mandibular contouring, trauma, dental extractions, periodontal disease, denture use, and previous facial or neck rejuvenation procedures should be documented. Preoperative photographs are particularly valuable in suspected iatrogenic cases. Symptoms of lower-lip incompetence, altered speech, drooling, sensory disturbance, scar tethering, and dissatisfaction with projection or contour should be recorded separately because they may indicate different anatomical problems [2,3,8,23,24].

Examination should be standardized in frontal, profile, and three-quarter views. The position of the chin pad relative to the inferior mandibular border, the depth and height of the labiomental and submental creases, lower-lip length, resting mandibular incisor exposure, lip competence, soft-tissue thickness, skin and fat excess, platysmal laxity, jowling, and cervicomental definition should be assessed. (Figure 4) Palpation helps distinguish a sharp tethered crease from generalized laxity and can identify implant edges, scar adherence, or an abrupt step between bone and soft tissue. Occlusion and skeletal projection must be evaluated before attributing the deformity to the soft-tissue envelope alone [2,8,13,19,23].

### 5.2. Dynamic Assessment

Static examination is insufficient when dynamic ptosis or mentalis dysfunction is suspected. Standardized photographs or video should be obtained at rest, during gentle lip closure, maximal lower-lip elevation or pouting, and smiling. The examiner should document whether the chin pad elevates normally, becomes flattened against the symphysis, descends below the mandibular border, develops asymmetric dimpling, or produces lower-lip distortion. Recording identical camera position, focal length, head posture, and facial tasks improves longitudinal comparison [2,14,25,27].

### 5.3. Objective Measurements and Patient-Reported Outcomes

No validated diagnostic threshold currently defines the severity of chin ptosis. Nevertheless, reproducible within-patient measurements can improve documentation and outcome assessment. Useful variables include the vertical position of the soft-tissue supramentale and soft-tissue pogonion relative to a stable horizontal or mandibular reference, lower-lip length, resting lower-incisor exposure, mentolabial angle and fold depth, the distance of the lowest point of the chin pad below the inferior mandibular border, chin projection, and the cervicomental angle. Lateral cephalometry or CBCT is appropriate when skeletal disproportion, alveolar resorption, previous osteotomy, implant position, or bone loss is relevant. Three-dimensional stereophotogrammetry or calibrated surface scanning can quantify contour and volume change, although such methods have not yet been standardized specifically for chin ptosis [23,29].

Ultrasonography can demonstrate mentalis thickness, depth, symmetry, and gross continuity and may be useful before revision surgery or muscle-directed injection [9]. Patient-reported outcomes should complement, rather than replace, anatomical measurements. The FACE-Q Satisfaction with Chin scale is a 10-item patient-reported outcome measure addressing chin size, width, shape, appearance from different angles, and appearance in photographs. Raw responses are converted using the instrument’s scoring table to a 0–100 score, with higher scores representing greater satisfaction [30,31]. Because the scale measures satisfaction rather than structural correction, FACE-Q results should be interpreted alongside standardized photographs, functional assessment, and objective measurements.

## 6. Management of Chin Ptosis

### 6.1. Surgical Treatment

#### 6.1.1. Restoration of the Mentalis Attachment and Chin-Pad Suspension

When true chin-pad descent results from detachment or inferior displacement of the mentalis, the anatomical objective is to restore the muscle close to its original cephalic origin rather than merely tighten the skin. Established correction requires release of scarred or malpositioned tissues, identification of viable muscle, symmetric advancement, and secure fixation using transosseous sutures, anchors, or another stable construct. Muscle reefing may help recreate the labiomental fold, but excessive tension can cause a deep fold, bunching, dimpling, lower-lip retraction, or oral incompetence [2,7,8,24]. Precise primary reapproximation is preferable to revision: Chaushu et al. demonstrated better preservation of lower-lip length, incisor exposure, and soft-tissue supramentale position after accurate reattachment, while Zhang et al. reported no witch’s chin deformities with a no-degloving technique compared with six after conventional degloving [10,23].

#### 6.1.2. Surgical Treatment Based on Soft-Tissue Reduction and Cervicofacial Rejuvenation

In contrast to the procedures described above, which correct chin ptosis by reattaching or cephalically resuspending the mentalis muscle, a separate group of operations targets redundant tissues, an abnormally deep submental crease, generalized cervicofacial laxity, or a mismatch between the osseous chin and its soft-tissue envelope. These methods are most appropriate when the dominant abnormality is excess skin or fat, soft-tissue overprojection, loss of cervicomental definition, or a combined aging-related deformity rather than isolated detachment of the mentalis muscle [2,20]. The most explicitly non-suspensory procedure was described by Feldman, who advocated direct excision of redundant chin skin, subcutaneous fat, and, when necessary, muscle, without attempting to elevate or reposition the remaining ptosis-prone tissues. A deep submental crease could be excised simultaneously, whereas deficient anterior projection could be corrected with a stable alloplastic implant. This approach is particularly applicable to a bulky or anteriorly overprojecting chin pad and to selected cases of dynamic soft-tissue excess, although it entails an external scar and irreversible tissue removal [20]. Other local procedures redistribute tissue rather than restore the mentalis attachment. Loeb advanced a local fat flap into the retracted submental fold, whereas Field used a suprahyoid fat flap for a scar-related witch’s chin deformity [28,32]. Peterson and Lesavoy similarly described dermal or dermal-fat flaps, sometimes combined with platysma plication or chin augmentation, to obliterate the submental crease and smooth the transition between the chin and neck [3,19]. These flap-based procedures primarily correct an illusory, crease-related, or volume-deficient component and should not be regarded as equivalent to restoration of a detached chin pad.

When chin ptosis coexists with generalized aging of the lower face and neck, direct treatment of the mental region may be incorporated into rhytidectomy. Pontes extended the routine face-lift dissection medially across the entire mental region, allowing the chin skin to be mobilized in the same anatomical plane as the facial and cervical flap and subsequently redraped into a more favorable position. The technique was evaluated in 500 rhytidectomy patients and could be combined with additional procedures addressing the neck, face, or chin [13]. Peterson likewise approached the senile chin deformity as part of face-lift surgery, combining correction of the submental fold with dermal-fat flaps, platysmal plication, and, when skeletal projection was inadequate, placement of a chin implant [19]. These reports suggest that lower facelift and neck lift are most useful when chin ptosis accompanies diffuse skin laxity, jowling, platysmal laxity, and loss of cervicomental definition. However, a conventional lateral face-lift that does not extend into the central mental region should not be expected to correct focal chin-pad ptosis reliably [1,13]. Supraplatysmal liposuction or open lipectomy may be added when genuine adipose excess is present, but fat removal alone neither restores a descended chin pad nor corrects loss of skeletal support and should therefore be regarded as an adjunct rather than definitive treatment for structural chin ptosis [1,2].

The role of facelift in chin ptosis is therefore primarily determined by the extent of the associated cervicofacial aging changes. In patients with lower-face laxity, jowling, cervical skin excess, platysmal banding, and an obtuse cervicomental angle, an extended lower facelift or combined face-and-neck lift can improve the position and contour of the chin by mobilizing and redraping the facial and cervical soft-tissue envelope. Pontes emphasized that the dissection must extend medially into the mental region if the chin skin is to be repositioned effectively; a purely lateral facelift does not adequately address central premental laxity [13]. Peterson similarly incorporated correction of the senile chin into facelift surgery and recommended combining soft-tissue treatment with platysmal plication, correction of the submental fold, and chin augmentation when skeletal projection was deficient [19]. These principles are consistent with contemporary cervicofacial rejuvenation techniques, in which the superficial musculoaponeurotic system and platysma are repositioned to improve lower-face definition and cervical contour. However, facelift should not be considered a substitute for direct correction of isolated mentalis detachment or a discrete descended chin pad. When the ptosis is focal and the surrounding skin and neck are relatively preserved, a targeted mentalis or chin-pad procedure is more appropriate. Conversely, when chin ptosis forms part of a broader aging deformity, facelift and neck lift may provide more harmonious correction than an isolated chin operation [1,2,13,19].

#### 6.1.3. Skeletal and Combined Procedures

More extensive procedures should be selected according to the individual components of the deformity. Bernardi et al. proposed a progressive three-step strategy directed at premental soft-tissue ptosis, the depth of the submental fold, and mandibular hyperprojection; one, two, or all three components were treated, and subsequent steps could be omitted once satisfactory correction had been achieved [33]. When a prominent osseous chin coexists with an excessive soft-tissue envelope, Zide and Longaker described submental ostectomy combined with simultaneous soft-tissue excision, thereby reducing skeletal projection while limiting the residual redundancy and secondary ptosis that may follow bone reduction alone [34]. At the less invasive end of the spectrum, de la Torre et al. introduced a U-shaped polypropylene suture placed through a small intraoral incision to gather and elevate the soft-tissue chin pad above the inferior mandibular border [5]. More recently, Sozer et al. described subcutaneous and subperiosteal mobilization followed by stabilization of the repositioned chin envelope with a 5–0 polypropylene surgical net, generally performed as part of comprehensive face and neck rejuvenation. In their retrospective series of 20 women, FACE-Q Satisfaction with Chin scores increased from 34 to 86 at one year, and no major complications were reported [1]. Although this technique involves dissection beneath the mentalis muscle, it stabilizes the chin envelope en bloc rather than reattaching the muscle origin and may therefore be classified as a separate soft-tissue fixation strategy. Nevertheless, the available evidence for most of these procedures remains limited to technical descriptions, small case series, and retrospective studies. Consequently, treatment should be tailored to the predominant anatomical abnormality, and combined correction of skin, fat, platysma, submental crease, and skeletal projection may be required instead of relying on a single universal technique [1,2,33,34].

Recent publications reinforce the need to coordinate skeletal correction with management of the soft-tissue envelope, although most do not study true chin ptosis directly. Guignardat et al. described a soft-tissue genioplasty for patients with an acute lower lip–chin prominence angle or excessive sagittal soft-tissue prominence, with or without conventional osteotomy [35]. Haas et al. used three-dimensional analysis to compare conventional and minimally invasive genioplasty approaches; their findings support the hypothesis that limiting soft-tissue detachment may improve predictability in the mentolabial region, but the study did not evaluate a chin-ptosis cohort [36]. Teuber Lobos et al. reported 24 patients treated for a long, hyperprojected chin using symphyseal resection with soft-tissue suspension; no major complications were reported and the mean FACE-Q score was 96.02, but the series represented level-IV evidence in a macrogenia population rather than direct evidence for isolated ptosis [37]. These studies are therefore relevant to prevention and combined deformities but should not be interpreted as proof of efficacy for every subtype of chin ptosis (Table 3).

### 6.2. Nonsurgical Management

#### 6.2.1. Botulinum Toxin Type A

BoNT-A may be considered when excessive or abnormally distributed mentalis activity is the dominant finding and the chin pad remains structurally supported. Its evidence base concerns hyperactive or high-riding mentalis muscle and cobblestone chin more than true structural ptosis. Hsu and Frankel demonstrated that chemodenervation can move the soft-tissue pogonion inferiorly and redistribute volume toward the lower chin [25]. This effect may be desirable in selected patients with excessive superior pull but may worsen an already descended pad. Treatment should therefore follow dynamic examination and, where anatomy is unclear, ultrasonographic localization. Excess dose, superficial placement, asymmetry, or diffusion into adjacent lower-lip depressors can cause lower-lip ptosis, oral incompetence, smile asymmetry, speech difficulty, or paradoxical bulging [9,26,27].

#### 6.2.2. Hyaluronic Acid and Other Volumizing Treatments

Hyaluronic acid filler can increase anterior projection, camouflage a deep crease, or smooth a localized contour depression when deficient volume contributes to the appearance. Randomized studies and systematic reviews show favorable short-term outcomes for chin retrusion and augmentation [38,39,40,41,42]. These data are indirect: filler does not reattach the mentalis, restore a disrupted periosteal support system, or permanently reposition a descended chin pad. Risks include edema, bruising, tenderness, asymmetry, nodules, infection, contour irregularity, and rare vascular occlusion or tissue necrosis. Filler should therefore be described as temporary camouflage or an adjunct for selected projection- or volume-deficient cases, not as definitive treatment for true structural chin ptosis [2,39,41,42].

#### 6.2.3. Scope and Limitations of Nonsurgical Treatment

Evidence reviewed here does not establish energy-based skin tightening, submental fat-reduction procedures, or thread-based suspension as definitive treatments for true structural chin ptosis. Loss of mentalis attachment or skeletal support requires separate anatomical assessment; reduction in fat or tightening of skin should not be assumed to restore these structures [1,2]. On this basis, the authors propose restricting nonsurgical management to selected mild, component-specific deformities, patients who decline surgery, or adjunctive camouflage after structural correction. Evidence concerning mentalis hyperactivity or deficient projection should be explicitly identified as indirect evidence rather than proof of correction of a descended chin pad [25,38,39].

## 7. Complications and Risk Reduction

Complications should be reported according to the anatomical component treated. Muscle-related problems include recurrent descent, incomplete correction, overcorrection, asymmetry, an excessively deep or displaced labiomental fold, bunching, dimpling, lower-lip retraction, altered incisor exposure, and oral incompetence. Extensive or repeated dissection can produce bleeding, hematoma, infection, wound dehiscence, scar tethering, mental nerve injury, dysesthesia, or difficulty identifying viable muscle. Direct excision and local flap procedures can leave a visible submental scar, dog ears, flap irregularity, contour depression, or irreversible under- or over-resection. Skeletal procedures add the risks of unfavorable contour, relapse, hardware or implant problems, bone resorption, and mismatch between the reduced bony framework and the residual soft-tissue envelope [2,3,10,14,20,23,24,34,41,42] (Table 4).

## 8. Discussion

The central finding of this review is that chin ptosis is a mechanism-dependent deformity rather than a single morphological diagnosis. The four-category classification used here derives from Garfein and Zide and remains clinically useful because it separates an apparent crease-related deformity, non-iatrogenic loss of support, postoperative mentalis disruption, and animation-dependent descent [2]. Its principal limitation is that the categories are not mutually exclusive. For this reason, etiological classification should be followed by a component-based assessment of skeletal projection, dentoalveolar support, mentalis position and function, crease morphology, soft-tissue volume, skin and platysmal laxity, and dynamic change.

The clarification of “developmental” ptosis is especially important. In the original framework, the term refers mainly to progressive, non-iatrogenic loss of mandibular and dentoalveolar support associated with periodontal disease, dentures, dental bone removal, or long-term edentulism, rather than to a congenital malformation [2,11,12,17]. Generalized senile laxity is related but not identical: aging may add skin redundancy, fat redistribution, platysmal laxity, and loss of cervicomental definition without being the primary cause of inferior mentalis displacement. Distinguishing these mechanisms prevents inappropriate reliance on facelift, skin excision, or skeletal augmentation alone.

Among the available interventions, the most direct clinical evidence supports preservation or accurate reattachment of the mentalis during genioplasty. Chaushu et al. demonstrated measurable differences in lower-lip and supramentale position after precise reattachment, and Zhang et al. reported fewer witch’s chin deformities with a no-degloving approach [10,23]. By contrast, much of the literature on local flaps, excisional correction, mentopexy, suture suspension, and combined skeletal–soft-tissue procedures consists of technical reports or small retrospective series. Sozer et al. reported a marked improvement in FACE-Q Satisfaction with Chin after surgical-net fixation, but the cohort comprised only 20 women, had no control group, and underwent the procedure within broader facial rejuvenation [1]. The improvement is encouraging but cannot establish superiority over anatomical muscle repair or define the most appropriate ptosis subtype.

Recent studies broaden the reconstructive options but also illustrate the problem of indirect evidence. Soft-tissue genioplasty, minimally invasive genioplasty, and symphyseal resection with soft-tissue suspension provide useful principles for limiting detachment and coordinating the bony framework with the soft-tissue envelope [35,36,37]. However, these studies primarily concern mentolabial contour, orthognathic surgery, or long and hyperprojected chins. Similarly, systematic reviews of implants, osteotomy, and injectable augmentation describe outcomes and complications of chin enhancement rather than structural chin ptosis [41,42]. Their findings should inform skeletal planning and consent, but they should not be cited as direct proof that a technique corrects a detached or descended chin pad.

The same distinction is essential for nonsurgical management. BoNT-A evidence concerns mentalis hyperactivity and demonstrates that weakening the muscle can itself move the soft-tissue pogonion inferiorly [25,26,27]. Filler evidence concerns projection deficiency and retrusion [38,40,41,42]. Both modalities can improve a selected component or camouflage a mild deformity, but neither restores a detached muscle origin. This separation between direct and indirect evidence should be maintained throughout future studies and clinical counseling.

The proposed algorithm translates these findings into practice. An exaggerated crease with preserved mentalis function is treated differently from postoperative muscle displacement; animation-dependent descent requires dynamic assessment; and static loss of support requires evaluation of the skeleton, dentition, skin, fat, and platysma. Mixed deformities may require combined correction, but each procedure should have a documented anatomical target. Standardized photography and video, reproducible measurements, explicit complication reporting, and FACE-Q assessment can then be used to evaluate whether the intended component was corrected rather than relying on a global subjective impression alone [2,23,29,31].

## 9. Limitations and Future Directions

The conclusions of this review must be interpreted in the context of several limitations. First, the available literature on chin ptosis is limited and highly heterogeneous. Most publications consist of anatomical studies, descriptions of individual operative techniques, expert opinions, case reports, and retrospective case series. Prospective comparative studies are scarce, and randomized studies comparing different surgical approaches are currently lacking. Although some authors have reported relatively large clinical experiences, patient-selection criteria, operative details, definitions of successful correction, follow-up periods, and methods of outcome assessment vary substantially between studies [5,13,20]. Consequently, direct comparison of the reported techniques and identification of a superior treatment method remain difficult.

A further limitation is the inconsistent terminology used to describe deformities of the chin. The terms chin ptosis, witch’s chin, senile chin deformity, deep submental crease, and chin-pad descent have sometimes been used interchangeably, despite representing different anatomical abnormalities. In particular, true descent of the chin pad caused by mentalis detachment should be distinguished from illusory ptosis related to a deep submental crease, age-related soft-tissue redundancy, dynamic displacement during facial animation, and inadequate or excessive skeletal projection [2]. Failure to differentiate these conditions may partly explain the variability in treatment selection and reported outcomes.

The methodological limitations of the available studies also restrict the strength of the conclusions. Standardized preoperative and postoperative photographic documentation is not consistently provided, dynamic assessment is rarely reported, and objective measurements of chin-pad position, submental crease depth, and cervicomental contour are generally absent. Moreover, validated patient-reported outcome measures have only recently been introduced into research on chin ptosis. Long-term information concerning recurrence, mentalis dysfunction, contour irregularities, scar quality, sensory disturbance, and the stability of skeletal and soft-tissue correction remains insufficient [1,5,10,14,23].

The methodological limitations of the available studies also restrict the strength of the conclusions. Standardized preoperative and postoperative photographic documentation is not consistently provided, dynamic assessment is rarely reported, and objective measurements of chin-pad position, submental crease depth, and cervicomental contour are generally absent. Moreover, validated patient-reported outcome measures have only recently been introduced into research on chin ptosis. Although Sozer et al. reported improvement using the FACE-Q Satisfaction with Chin scale, their study was retrospective, included a small and demographically homogeneous cohort, and lacked a control group [1]. Long-term information concerning recurrence, mentalis dysfunction, contour irregularities, scar quality, sensory disturbance, and the stability of skeletal and soft-tissue correction remains insufficient.

The present review is also limited by its narrative design. Study selection and evidence synthesis were not conducted according to a registered systematic-review protocol, and no formal risk-of-bias assessment or quantitative meta-analysis was performed. In addition, the inclusion of older foundational reports was necessary because many established techniques have not been reassessed in contemporary comparative studies. Some historical procedures are therefore supported primarily by technical descriptions rather than standardized clinical evidence. Future research should prioritize prospective, multicenter studies employing uniform definitions and classification criteria. A standardized assessment protocol should incorporate examination at rest and during facial animation, evaluation of mentalis function, measurement of skeletal projection, assessment of the submental crease and cervicomental angle, and documentation of previous surgical disruption of the mentalis attachment. Three-dimensional surface imaging, standardized photography, cephalometric analysis, and validated patient-reported measures such as FACE-Q could improve the objectivity and comparability of treatment outcomes [2,23,29,30,31].

Comparative studies are particularly needed to evaluate direct mentalis reattachment, cephalic resuspension, suture-based chin-pad fixation, local dermal or dermal-fat flaps, direct soft-tissue excision, facelift-based redraping, and combined skeletal and soft-tissue procedures. The role of anatomical variation in determining surgical outcomes also requires further investigation, as differences in the degree of midline fusion and muscle development may influence the stability of mentalis repair and postoperative chin contour [9]. Particular attention should be given to procedures involving additional subperiosteal dissection or reduction in the bony chin, because both maneuvers may compromise soft-tissue support when they are not accompanied by adequate mentalis reconstruction or chin-pad stabilization [2,14]. The component-based algorithm proposed in this review should be regarded as a clinically reasoned framework rather than a validated decision rule. Future prospective studies should test its interobserver reliability, determine whether it improves patient selection, and evaluate outcomes after stratification by ptosis subtype and dominant anatomical component.

## 10. Conclusions

Chin ptosis is a heterogeneous deformity produced by different combinations of skeletal support loss, mentalis detachment or dysfunction, an abnormally deep submental crease, soft-tissue redundancy, and altered facial animation. The established categories of illusory, developmental, iatrogenic, and dynamic ptosis are clinically useful when they are understood as dominant mechanisms rather than mutually exclusive diagnoses.

Treatment should be selected according to the anatomical abnormality demonstrated on static and dynamic assessment. Structural mentalis displacement requires anatomical restoration; crease deformities require local correction; skeletal deficiency or excess requires skeletal planning; and diffuse skin, fat, or platysmal laxity may require soft-tissue reduction or cervicofacial rejuvenation. Nonsurgical methods can camouflage selected mild components but cannot replace reconstruction of a detached or unsupported chin pad.

No operative technique can be recommended universally, and the current evidence is insufficient to identify a superior approach. More reliable comparisons will require standardized diagnostic criteria, reproducible measurements, validated patient-reported outcomes, consistent complication reporting, and long-term prospective studies stratified by chin-ptosis subtype.

## Figures and Tables

**Figure 1:**
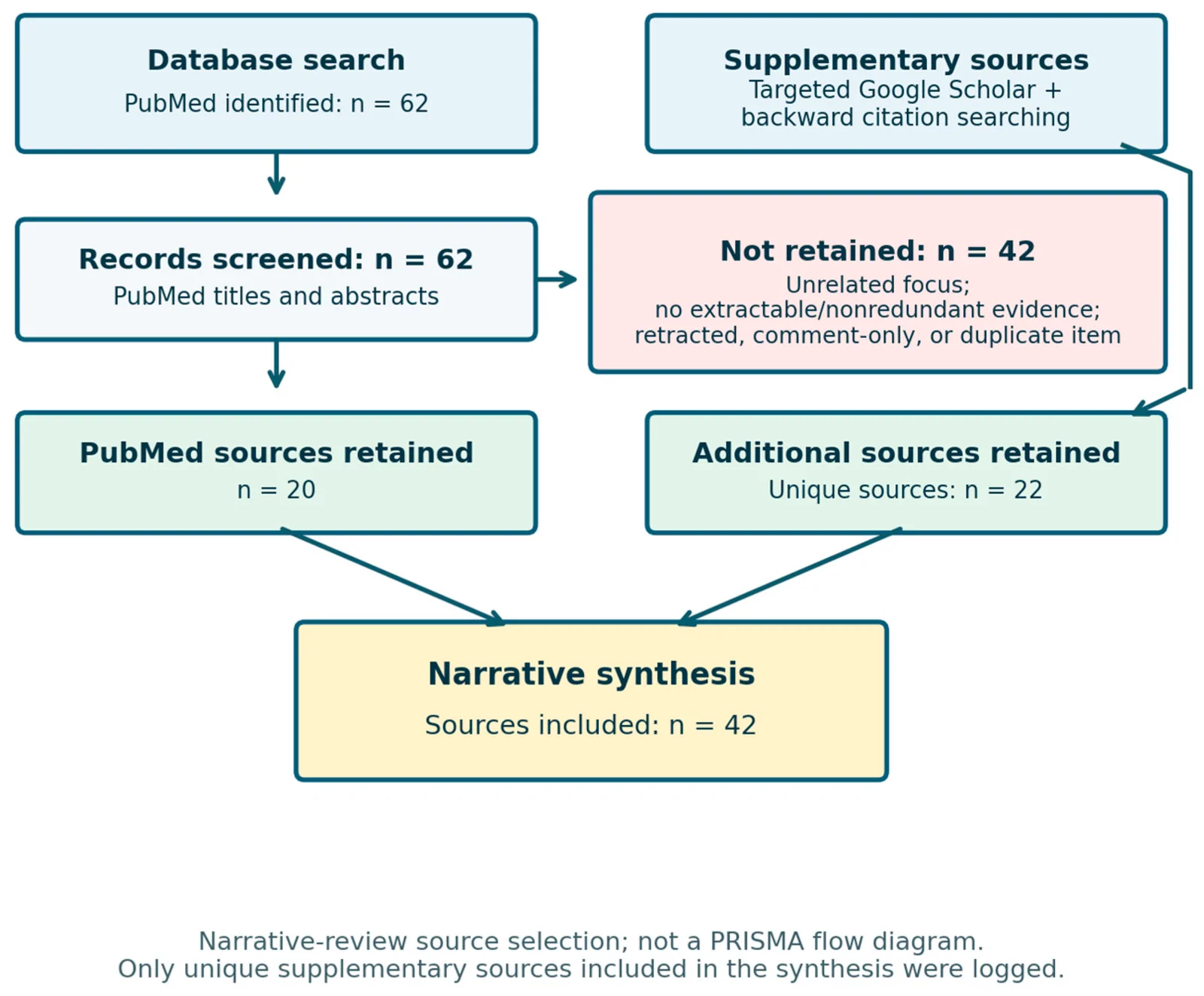
Source-selection flow for the narrative review. The diagram reports the verified PubMed yield and the unique additional sources retained through targeted Google Scholar and backward citation searching. It documents transparent source selection but does not imply full PRISMA compliance.

**Figure 2:**
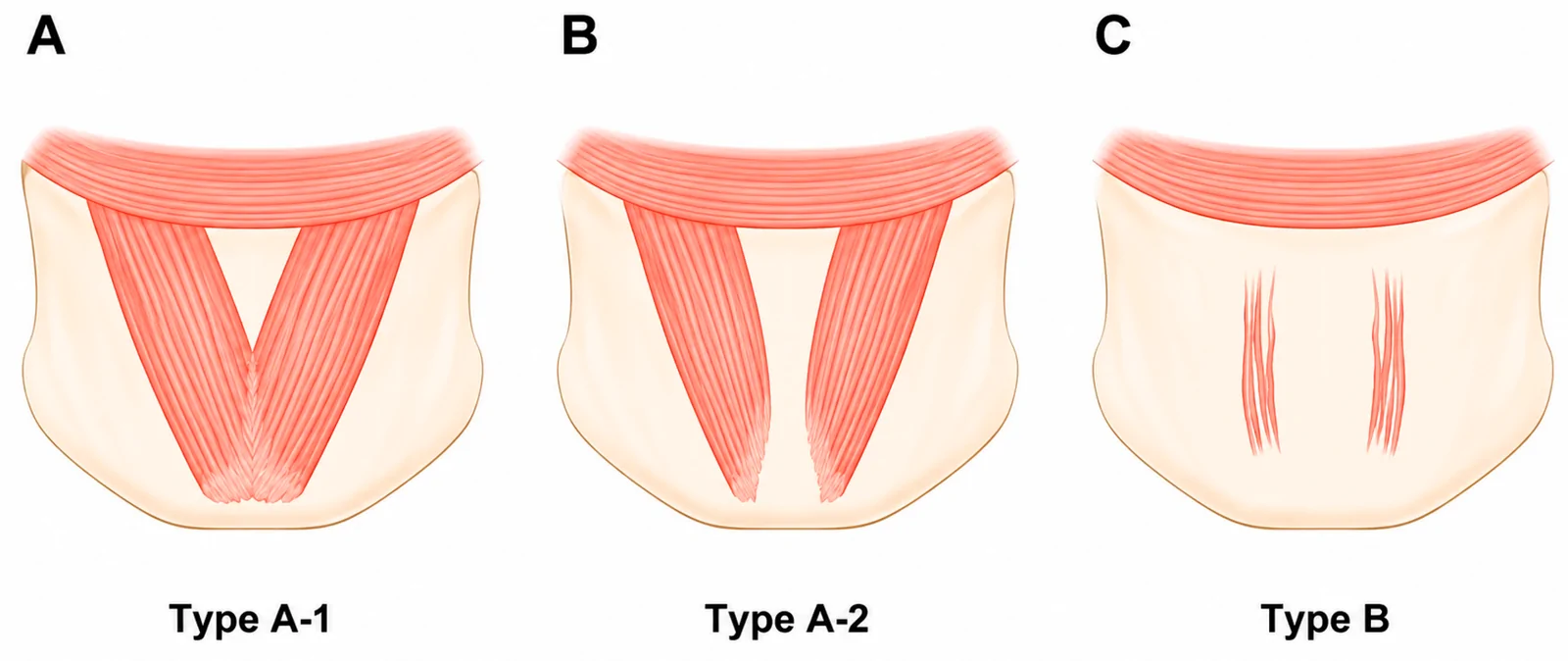
(**A**) Type A-1, two bilateral mentalis muscles merged with each other. (**B**) Type A-2, the two mentalis muscles were separated. (**C**) Type B, mentalis muscle was flat and comprising only a few muscle fibers.

**Figure 3:**
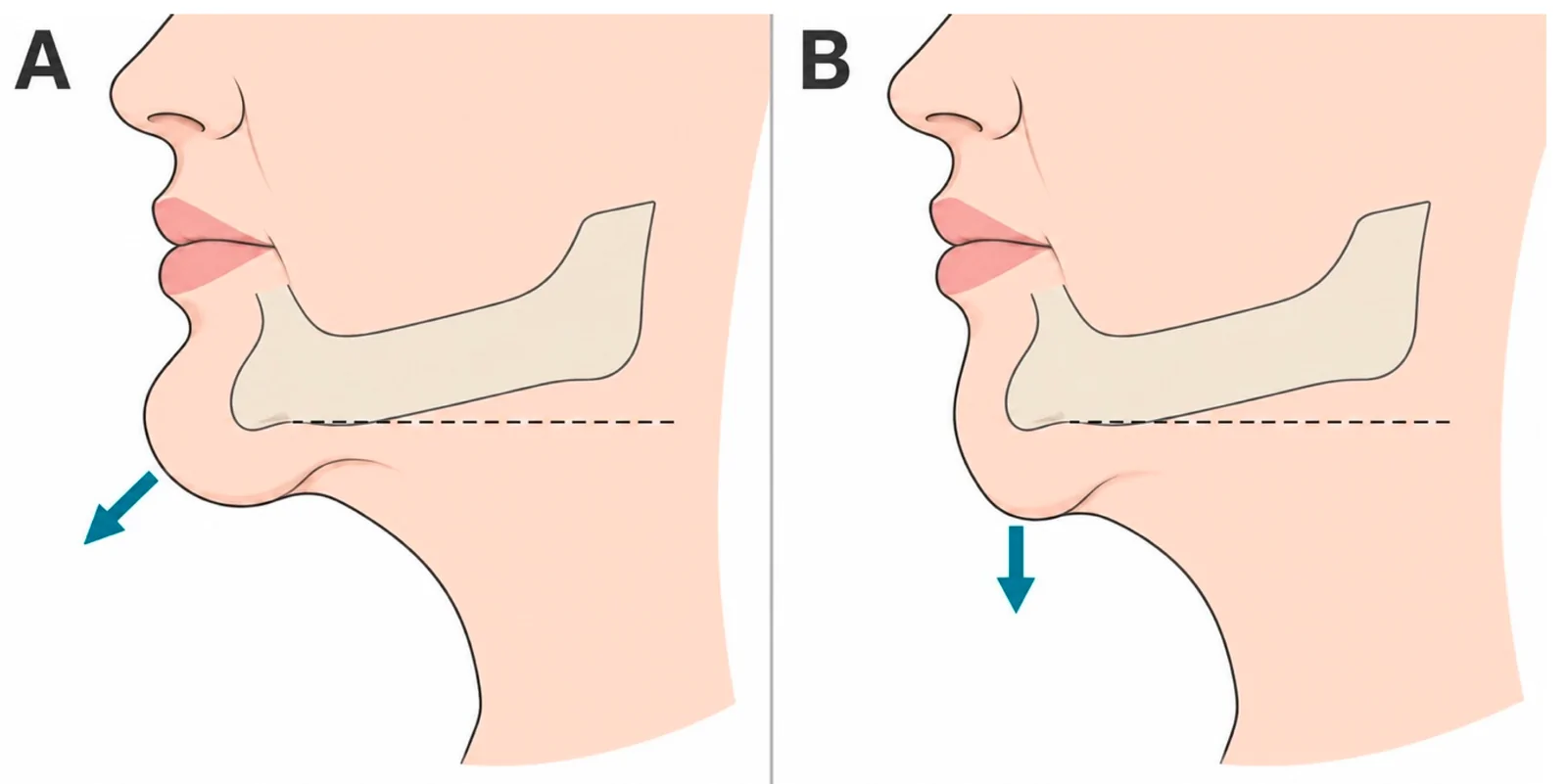
Schematic comparison between witch’s chin deformity and true chin ptosis. (**A**) Witch’s chin is characterized by inferior and anterior displacement of the premental soft-tissue pad, a pronounced submental crease, and frequently reduced underlying bony projection. (**B**) True chin ptosis represents predominantly inferior displacement of the presymphyseal soft tissues below the inferior mandibular border, without excessive anterior projection. The dashed line indicates the inferior mandibular border, and the arrows show the predominant direction of soft-tissue displacement [3,13].

**Figure 4:**
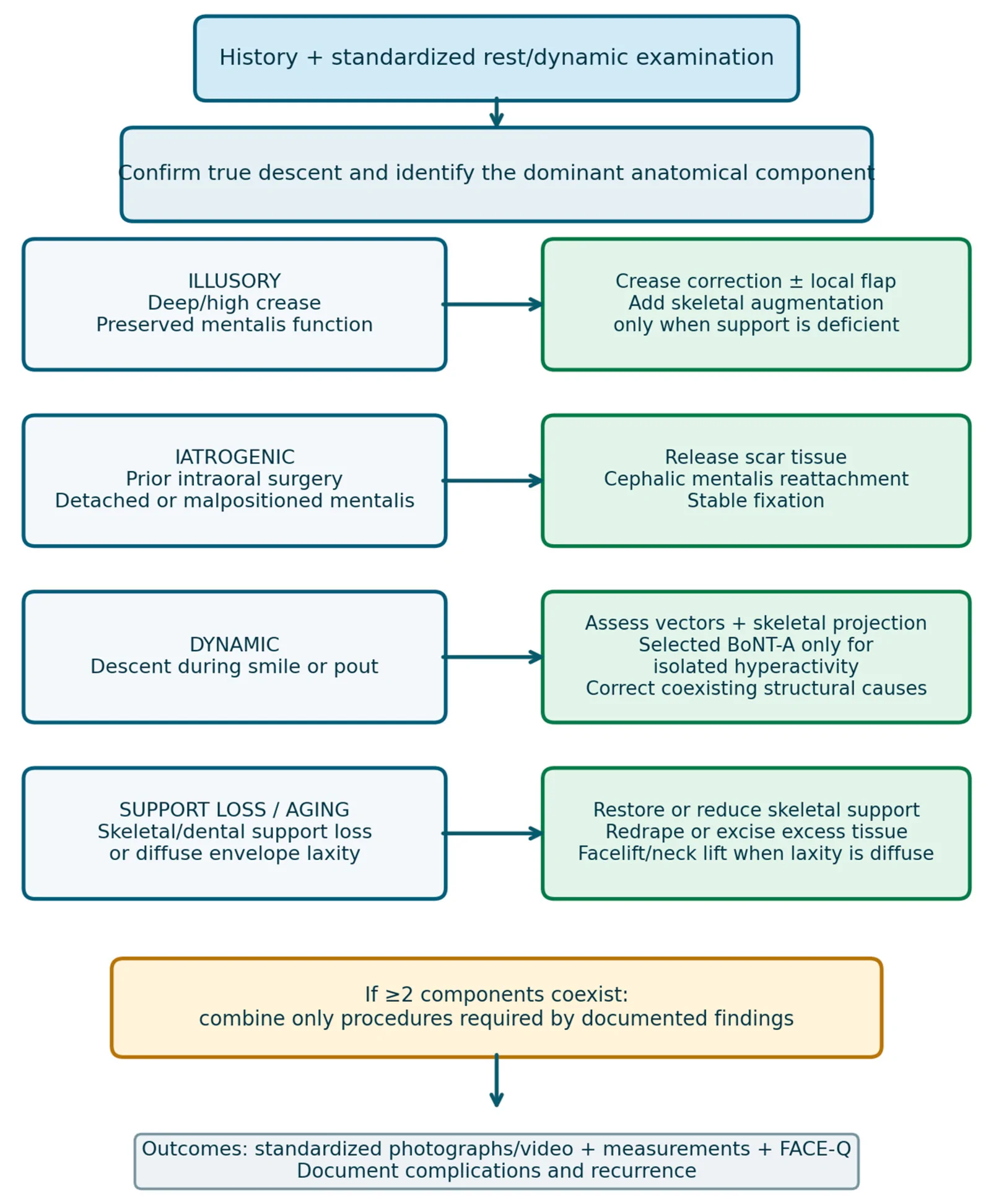
Component-based diagnostic and treatment algorithm for chin ptosis. The four etiological categories identify the predominant mechanism, while definitive treatment is selected according to the documented skeletal, muscular, crease-related, soft-tissue, and dynamic components.

**Table 1:** Documented search and supplementary source-identification strategies.

Source/Search Method	Reproducible Strategy	Coverage and Yield
MEDLINE via Pub-Med	(“chin ptosis” [Title/Abstract] OR “ptotic chin” [Title/Abstract] OR “witch’s chin” [Title/Abstract] OR “senile chin deformity” [Title/Abstract] OR (“mentalis muscle” [Title/Abstract] AND (anatomy [Title/Abstract] OR ptosis [Title/Abstract] OR reattachment [Title/Abstract] OR resuspension [Title/Abstract] OR genioplasty [Title/Abstract])) OR (“chin pad” [Title/Abstract] AND (ptosis [Title/Abstract] OR descent [Title/Abstract] OR suspension [Title/Abstract])) OR (“submental crease” [Title/Abstract] AND (chin [Title/Abstract] OR correction [Title/Abstract]))).	Database inception–6 October 2026; 62 records retrieved and screened.
Targeted Google Scholar searching	Separate phrase searches: “chin ptosis”; “ptotic chin”; “witch’s chin”; “senile chin deformity”; “mentalis muscle” AND (reattachment OR resuspension OR genioplasty); “chin pad” AND (descent OR suspension); “submental crease” AND correction.	Repeated 6 October 2026. Together with citation searching, 22 unique additional sources were included.
Backward citation searching	Reference lists of retained articles and pertinent narrative or systematic reviews were examined for additional anatomical, historical, diagnostic, and treatment sources not retrieved by the PubMed strategy.	Completed 6 October 2026. Count reported jointly with targeted Google Scholar searching to avoid double counting

**Table 2:** Characterization and clinical implications of chin ptosis.

Type of Ptosis	Diagnostic Pattern and Objective Assessment	Dominant Mechanism	Management Direction and Principal Caveat
Illusory	Apparent descent created by a high or deep submental crease. Lower-lip position, intraoral sulcus, and mentalis movement are usually preserved. Assessment should include standardized profile photographs at rest and during animation, palpation of the crease, and evaluation of skeletal projection [2,3,19]	Crease abnormality and/or localized volume deficiency, sometimes accompanied by deficient skeletal projection [2,20].	Treatment may include crease excision or de-epithelialization and the use of a dermal, dermal-fat, or local fat flap when volume is deficient. Chin augmentation may be considered when inadequate skeletal projection contributes to the deformity. Mentalis resuspension alone does not correct a primarily crease-related deformity [3,19,20,21,28]
Developmental	No causative chin operation is identified. Contributing findings may include tooth loss, periodontal disease, denture use, alveolar or symphyseal bone resorption, and age-related soft-tissue laxity. Assessment should include dental and prosthetic history, occlusion, facial profile, lower-face and neck examination, and cephalometry or CBCT when skeletal loss is clinically relevant [11,12,17].	Non-iatrogenic loss of dental and skeletal support may cause inferior displacement of the mentalis origin. Aging can additionally produce skin redundancy, fat redistribution, platysmal laxity, and loss of cervicomental definition [2,11,13,19]	Dental and skeletal support should be stabilized first. Augmentation, correction of the submental crease, soft-tissue redraping, direct excision, or cervicofacial rejuvenation may then be selected according to the residual deformity. Dental rehabilitation alone does not reverse established muscle displacement or soft-tissue redundancy [2,13,19].
Iatrogenic	The deformity develops after intraoral genioplasty, placement or removal of a chin implant, or repeated chin surgery. Findings may include an altered labiogingival sulcus, increased lower-lip length, altered incisor show, scar tethering, bunching, or static descent of the chin pad. Preoperative photographs should be reviewed, and the chin should be examined both at rest and during animation. CBCT or ultrasound may be used selectively [2,8,23,24].	Transection, devascularization, inferior healing, or inadequate reattachment of the mentalis muscle may produce loss of soft-tissue support. Removal of an implant or excessive bone reduction may further increase the mismatch between skeletal support and the soft-tissue envelope [8,10,23].	Treatment generally requires release of scarred tissues followed by anatomical cephalic reattachment or resuspension of the mentalis muscle with stable fixation. Associated skeletal or soft-tissue-envelope deficiencies should be corrected simultaneously. Revision is less predictable when the muscle is shortened, attenuated, denervated, or inseparable from scar tissue [2,8,10,23,24].
Dynamic	The deformity may be minimal at rest, while the chin pad flattens or descends during smiling, pouting, or forceful lip closure. Assessment should include standardized video recordings or sequential photographs at rest and during lip closure, pout, and smile, together with evaluation of smile vectors and skeletal projection [2,14,25,27]	Abnormal muscular vectors and compression of the chin pad against the mandibular symphysis may produce dynamic descent. The deformity can be accentuated by excessive skeletal projection, mentalis hyperactivity, or previous reduction procedures [14,20,25].	Treatment should address the responsible component. Selected muscle modulation may be considered for isolated hyperactivity, whereas soft-tissue fixation, skeletal reduction, or correction of skeletal projection may be required when structural factors coexist. Botulinum toxin may worsen lower-lip position or redistribute chin volume inferiorly and should therefore be used cautiously [14,20,25,27].

**Table 3:** Comparison of the studies evaluating chin ptosis etiology and treatment.

Study	Design/Sample	Anatomical Target and Technique	Main Reported Finding	Direction and Limitation
Chaushu et al., 2001 [23].	Comparative cohort n = 21	Precise mentalis reattachment during advancement genioplasty	Better preservation of lower-lip length, incisor show, and soft-tissue supramentale; control group showed 2.14 mm inferior supramentale displacement.	Direct evidence for prevention of postoperative descent; small, nonrandomized cohort.
de la Torre et al., 2004 [5]	Technical report/limited clinical series	U-shaped polypropylene suture suspension of the chin pad	Minimally invasive elevation above the inferior mandibular border.	Direct but low-level evidence; limited standardized outcomes and comparative data.
Torrealba et al., 2017 [14]	Technical report	Submental crease/soft tissue correction combined with pogonion ostectomy	Addresses dynamic ptosis associated with macrogenia and excess soft tissue.	Direct but restricted clinical evidence; mentalis detachment and reduced skeletal support may create additional risk.
Zhang et al., 2021 [10]	Retrospective comparative study; n = 50	No-degloving osseous genioplasty	Witch’s chin occurred in 0 patients after no-degloving versus 6 after conventional degloving; operative time was similar.	Direct evidence for prevention; nonrandomized design.
Sozer et al., 2025 [1]	Retrospective series; n = 20 women	Subcutaneous/subperiosteal mobilization and polypropylene surgical net	FACE-Q Satisfaction with Chin increased from 34 to 86 at 1 year; no major complications reported.	Direct but level-IV evidence; no control group, homogeneous cohort, and concurrent rejuvenation procedures.
Guignardat et al., 2025 [35]	Technical description	Soft-tissue genioplasty with or without osteotomy	Proposed for an acute lower lip–chin angle or excessive sagittal soft-tissue prominence.	Indirect evidence; not a comparative chin-ptosis study.
Haas et al., 2025 [36]	Retrospective 3D cohort study	Minimally invasive versus conventional genioplasty	Less soft-tissue detachment may improve predictability of mentolabial response.	Indirect evidence; orthognathic/genioplasty cohort rather than diagnosed ptosis.
Lobos et al., 2026 [37]	Retrospective series; n = 24	Symphyseal resection with soft-tissue suspension	No major complications; mean FACE-Q score 96.02.	Indirect level-IV evidence in long/hyperprojected chins; no comparison group.

**Table 4:** Complications, mechanisms, preventive measures.

Intervention/Component	Potential Complications	Risk-Reduction Principles
Mentalis reattachment or suspension	Recurrence, asymmetry, dimpling, bunching, deep labiomental fold, lower-lip retraction or incompetence, altered incisor show [2,7,8,23,24]	Identify viable muscle; release scar adequately; use symmetric, secure cephalic fixation; avoid excessive tension; assess lip function before closure [2,7,8,23,24].
Local flap or direct excision	Visible scar, dog ears, contour depression, flap irregularity, under- or over-resection, wound problems [3,19,20].	Match the procedure to crease depth and tissue excess; conservative staged excision when uncertainty exists; meticulous scar placement and closure [3,19,20].
Genioplasty, ostectomy or implant procedure	Mental nerve injury, implant/hardware problems, infection, bone resorption, skeletal–soft-tissue mismatch, secondary ptosis [10,34,41,42]	Limit degloving; protect the mental nerves; plan bone and envelope changes together; precisely reattach the mentalis [10,34,41,42]
Facelift/neck lift or lipectomy	Hematoma, contour irregularity, skin compromise, residual central ptosis, over-resection of fat [1,2,13,19].	Extend release into the mental region only when indicated; preserve vascularity; treat focal mentalis detachment separately; remove fat conservatively [1,2,13,19].
BoNT-A	Lower-lip ptosis, oral incompetence, smile asymmetry, speech difficulty, paradoxical bulging, inferior volume redistribution [9,26,27].	Confirm a muscle-dominant indication; use conservative, anatomically targeted dosing; avoid adjacent lower-lip depressors [9,25,26,27]
Hyaluronic acid filler	Edema, bruising, tenderness, nodules, infection, asymmetry, contour irregularity, vascular occlusion or necrosis [38,39,41,42].	Treat projection/volume deficiency rather than structural descent; use anatomical planes and conservative volumes; maintain vascular-complication preparedness [38,39,41,42].

## Data Availability

No new data were created or analyzed in this study. Data sharing is not applicable to this article.
